# Mitochondrial ribosomal proteins in metastasis and their potential use as prognostic and therapeutic targets

**DOI:** 10.1007/s10555-024-10216-4

**Published:** 2024-10-01

**Authors:** Jasmine M. Bacon, Johanna L. Jones, Guei-Sheung Liu, Joanne L. Dickinson, Kelsie Raspin

**Affiliations:** 1 Menzies Institute for Medical Research, University of Tasmania, Hobart, TAS Australia; 2 Centre for Eye Research Australia, Royal Victorian Eye and Ear Hospital, East Melbourne, Victoria, Australia; 3 Ophthalmology, Department of Surgery, University of Melbourne, East Melbourne, Victoria, Australia

**Keywords:** Cancer, Metastasis, Mitochondrial ribosomal proteins, Prognostic targets, Therapeutic targets

## Abstract

The mitochondrion is an essential cell organelle known as the powerhouse of the cell. Mitochondrial ribosomal proteins (MRPs) are nuclear encoded, synthesised in the cytoplasm but perform their main functions in the mitochondria, which includes translation, transcription, cell death and maintenance. However, MRPs have also been implicated in cancer, particularly advanced disease and metastasis across a broad range of cancer types, where they play a central role in cell survival and progression. For some, their altered expression has been investigated as potential prognostic markers, and/or therapeutic targets, which is the focus of this review. Several therapies targeting MRPs are currently approved by the Food and Drug Administration and the European Medicines Agency for use in other diseases, revealing the opportunity for repurposing their use in advanced and metastatic cancer. Herein, we review the evidence supporting key MRPs as molecular drivers of advanced disease in multiple cancer types. We also highlight promising avenues for future use of MRPs as precision targets in the treatment of late-stage cancers for which there are currently very limited effective treatment options.

**Supplementary Information:**

The online version contains supplementary material available at 10.1007/s10555-024-10216-4.

## An introduction to mitochondrial ribosomal proteins

The mitochondrion is an essential sub-cellular organelle that generates energy for cellular function. The mitochondrial proteome comprises 1,500 proteins, and over 99% of these are nuclear encoded, including the 82 mitochondrial ribosomal proteins (MRPs), some of which will be the focus of this review [1, 2]. MRPs are synthesised in the cytoplasm of the cell and transported to the mitochondria, where they assist in ribosomal assembly. The mitochondrial ribosome is essential for the translation of messenger RNA (mRNA) within the mitochondria, and, like other ribosomes, the mitochondrial ribosome comprises two subunits with distinct roles. The small 28S subunit, comprised of 30 proteins encoded by 32 small MRP (MRPS) genes, facilitates the interaction between mRNA and transfer RNA (tRNA) [3, 4]. The large 39S subunit, consisting of 52 proteins encoded by 50 large MRP (MRPL) genes, promotes the formation of peptide bonds [3, 4].

**Fig. 1:**
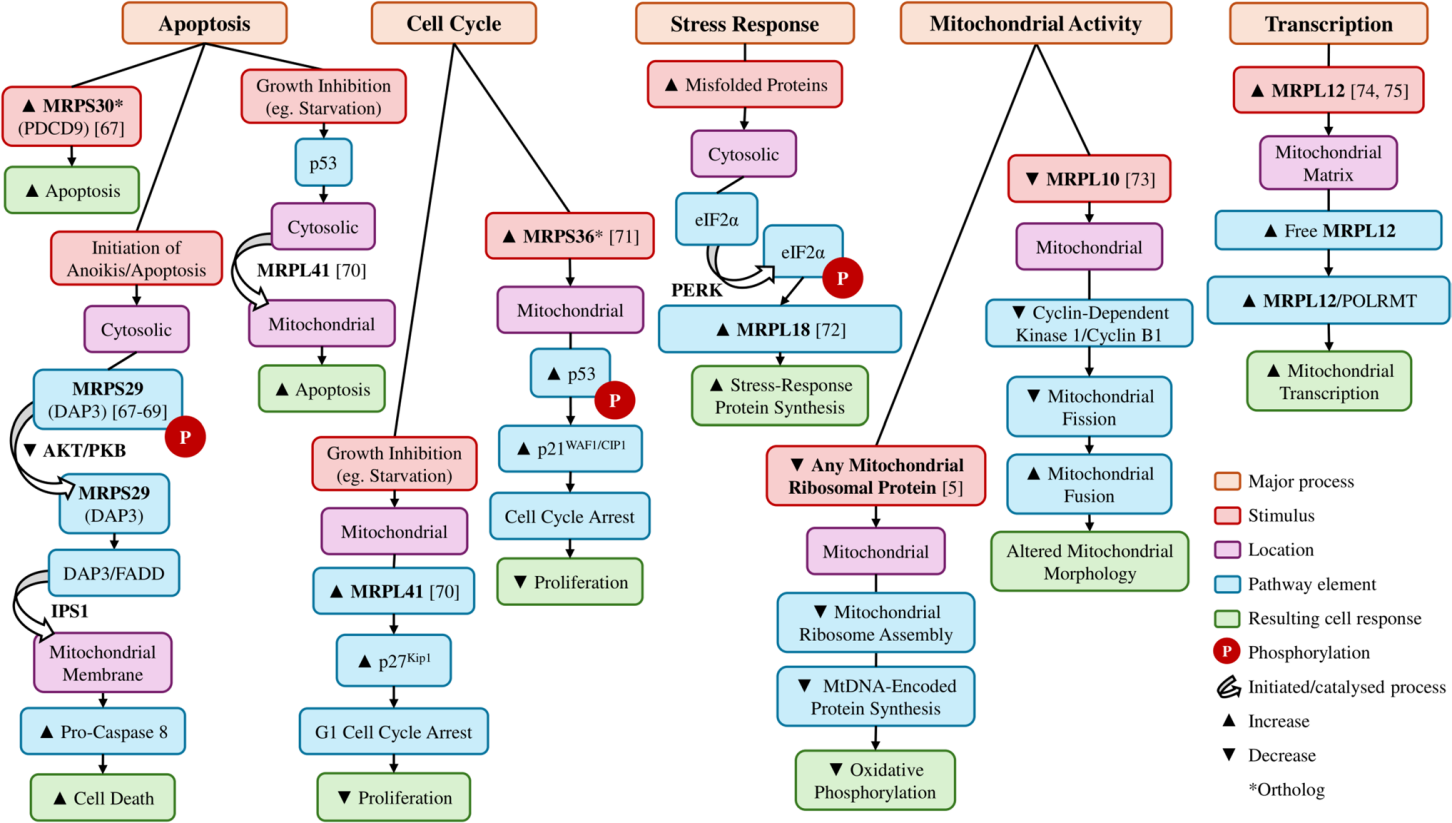
Normal cellular functions involving mitochondrial ribosomal proteins. Mitochondrial ribosomal proteins are involved in a wide range of cellular functions and responses, from apoptosis, cell cycle arrest, and stress response pathways to mitochondrial function and transcription. Although poorly characterised in humans, an increase in MRPS30 ortholog in mice fibroblasts increases apoptosis [6]. Cytosolic MRPS29 assists in activating anoikis in detached cells, through the extrinsic cell death activation pathway [6–8]. When cells are exposed to environmental changes inhibiting cell growth, MRPL41 stabilises p53 and supports its translocation into the mitochondria, where it induces apoptosis [9]. Additionally, under conditions inhibiting cell growth, MRPL41 also suppresses proliferation through G1 cell cycle arrest [9]. An increase in the MRPS36 ortholog in mice has been observed to promote the phosphorylation of p53 to induce cell cycle arrest and suppress tumour growth [10]. Cytosolic MRPL18 is increased as part of the cytosolic stress response, this then promotes increased synthesis of stress-response proteins [11]. Mitochondrial ribosomal proteins are required for mitochondrial-DNA encoded protein synthesis, which is critical for functional oxidative phosphorylation [5]. Decreased MRPL10 has been shown to decrease cyclin B1/Cdk1 activity, resulting in mitochondrial clustering and elongation [12]. Free MRPL12 within the mitochondrial matrix binds to POLRMT, an RNA polymerase, to increase promoter-dependent and -independent mitochondrial transcription [13, 14]

MRPs are involved in a variety of pathways crucial to cell function that extend beyond translation; from regulation of mitochondrial transcription and cell cycle to maintenance pathways, including apoptosis and cytosolic stress response (Fig. 1). As diverse pathways require MRP involvement, it is not surprising that dysfunction of these proteins has been implicated in various disorders, including cancer, neurodegenerative and metabolic disorders [5]. Without ribosomes, the mitochondria cannot produce key oxidative phosphorylation elements, and even reduced ribosome function can result in protein synthesis insufficient to maintain the basal metabolic rate [5]. Consequently, systemic disruption of MRP function can be lethal due to the critical role of mitochondrial ribosomes.

## Mitochondrial ribosomal proteins in cancer metastasis

MRPs have been implicated in tumorigenesis across a broad range of cancer types, with altered expression resulting in an increase in tumour cell heterogeneity, survival and progression to metastasis [15]. The process of metastasis, where cancer cells spread from the primary tumour site to distal organs, requires cells to adapt to vastly different and often changing environments. Specifically, the mitochondria of invasive cancer cells must undergo metabolic shifts to enable them to grow, survive and colonise at distal sites [16]. Traditionally, cancer cells are thought to switch from an epithelial state to acquire mesenchymal features, a key characteristic of cancer cell development (EMT; epithelial to mesenchymal transition). However, how cancer cells acquire these metabolic and mesenchymal features is poorly understood. In this review, we will discuss the current literature surrounding MRPSs and MRPLs in the context of metastatic cancer.

### Small mitochondrial ribosomal proteins

In total, five MRPSs have been implicated in metastasis of more than one cancer type, which equates to one seventh of all MRPSs (Table 1); whilst additional MRPSs have been associated with metastasis of only a single cancer type (one third of all MRPSs; see Supplementary Material). Of the MRPSs associated with more than one cancer type, *MRPS23* has been the most well-studied, however studies have found a conflicting role for the protein within and between cancer types [17–23].

**Table 1:** Overview of the small mitochondrial ribosomal proteins associated with metastasis in more than one cancer type

*MRPS*	Associated Cancer(s)	Associated Protein(s) or Gene(s)	Associated Pathway(s)	Association of Metastatic Traits with Patterns of Expression
*MRPS12*	Breast [24], Ovarian [25]	p53 [25]	Cell cycle [25], PI3K/Akt/mTOR [25], Immune infiltration [25]	▲ Drug resistance [24, 25], ▲ Recurrence [24, 25], ▲ Poor survival [25], ▲ Advanced stage [25]
*MRPS16*	Ovarian [26], Lung [27] (LUAD (Proximal-proliferative subtype) & LUSC)	HE4 [26]		▲ Poor survival [26, 27]
*MRPS18B*	Endometrial [28], Prostate [29], Breast [30]	E2F1 [28], Vimentin [28], TWIST2 [29], CXCR4 [29], p53 [30], NPAS2 [30], ROS1 [30]	PIP3/AKT [30], Oestrogen signalling [30], Cell cycle [30], Circadian rhythm [30]	▲ EMT [28, 29], ▲ Invasion [28, 29], ▲ Metastatic phenotype [29], ▲ Proliferation in endometrial cancer [28], ▼ Proliferation in prostate cancer [29]
*MRPS23*	Cervical [21], Breast [17–20, 22, 31] (Luminal B subtype [19]), Colorectal [32], Hepatocellular [23]	PDK2 [21], p21WAF1/CIP1 [18], Vimentin [18, 22], p53 [18, 22], HER2 [19], Ki67 [19], PRMT7-SETD6 [20], RIPK3 [22]	Cell cycle [21]	▼ Progression free survival [21], ▲ Proliferation [18–23], ▲ EMT [18, 22], ▲ Metastatic phenotype [19, 21, 23], ▼ Invasion in breast cancer [20], ▼ Metastatic phenotype in breast cancer [20], ▼ High grade in breast cancer [20], ▲ Methylation at k108me2 (SETD6) [20], ▼ Methylation at r21me1 (PRMT7) [20], ▲ Poor prognosis [32], ▲ Poor survival [23], ▲ Advanced stage [23]
*MRPS31*	Ovarian [26], Breast (Luminal A, Triple Negative) [30]	HE4 [26], ACADSB [30], CES1 [30], NPAS2 [30]	Fatty acid oxidation [30], BDNF [30], Folate biosynthesis [30], EGFR1 [30], PIP3/AKT [30], Hedgehog signalling [30], Wnt signalling [30], Oestrogen signalling [30]	▲ Poor survival [26], ▲ Poor progression free survival [26] ▲ Metastatic cell lines [30]

▲ Increased expression of MRPS is associated with the trait; ▼ Decreased expression of MRPS is associated with the trait. Abbreviations: LUAD, lung adenocarcinoma; LUSC, lung squamous cell carcinoma; EMT, epithelial to mesenchymal transition

An overview of the small mitochondrial ribosomal proteins associated with metastasis in only one cancer type can be found in Supplementary Table 1

#### *MRPS23*

Lyng *et al.* (2006) first identified an association between *MRPS23* expression and metastasis in cervical cancer [21]. Specifically, *MRPS23* was upregulated in cervical tumours with increased recurrence and lymph node metastases, and was associated with rapid growth and invasive capacity [21]. Moreover, a rat breast cancer model found similar results; long-term MRPS23 depletion reduced tumour growth and metastasis [18] and the authors suggested that MRPS23 may be involved in reversing the apoptotic pathway to assist metastatic progression [18]. Investigations in patient breast cancer samples have observed heightened *MRPS23* expression in high-grade, aggressive tumour samples [19], as well as an increase in tumour compared to normal tissue [22]. Although both studies did not reveal an association with patient survival, altered expression levels support a potential role of *MRPS23* in an aggressive breast cancer phenotype [19, 22]. Intriguingly, Oviya and colleagues (2021) found a breast cancer-specific MRPS23 isoform (or altered post-translational modification) that was expressed almost exclusively in breast cancer samples [22]. This altered post-translational modification was later defined to be methylation of two sites within *MRPS23* (K108me2, R21me1) and was observed to alter the expression of the resulting protein, with the rate of oxidative phosphorylation dependent on MRPS23 expression [20]. In contrast to the aforementioned studies, they also found that low MRPS23 was associated with high-grade disease and some metastatic traits [20]. Additionally, hypermethylation of *MRPS23* was associated with poor survival in breast cancer patients in a separate study [31], thus further supporting the suspected role of epigenetic regulation of *MRPS23* in breast cancer progression [31].

*MRPS23* also has prognostic value in colorectal cancer, where it was identified as one of 12 prognostic RNA binding proteins [32]. Although expression was increased in colorectal cancer, and was considered prognostic, the authors considered this expression profile to be low-risk (HR = 0.589) and did not elucidate the specific function of MRPS23 in colorectal cancer progression [32]. In hepatocellular carcinoma, increased MRPS23 expression is associated with larger, later stage tumours, and poor survival [23]. Despite this, knockdown has not been shown to effect the metastatic capability of hepatocellular carcinoma cells (unlike its effects in breast cancer), suggesting the change in expression may be a response to increased metastatic capacity, not causative [18, 23]. These results could also suggest that modifications to the expression of MRPS23 may only occur in, or only provide a survival advantage in certain cancer types, such as breast cancer.

#### *MRPS12*

Another MRPS associated with metastasis, which is a promising prognostic and therapeutic target is *MRPS12*. Increased expression has been consistently linked to metastatic traits as well as therapeutic resistance [24, 25]. Sotgia and colleagues (2017) investigated the association between nuclear-encoded mitochondrial-associated genes and high-risk estrogen receptor-positive breast cancer and found that increased expression of *MRPS12* was associated with tumour recurrence and tamoxifen-resistance [24], both of which are precursors to metastatic disease. Likewise, in ovarian cancer, the expression of *MRPS12* is also associated with recurrence and advanced disease stage [25]. Gene set enrichment analysis of 426 ovarian cancer tumours from The Cancer Genome Atlas (TCGA) revealed *MRPS12* overexpression was correlated with the activation of biological pathways such as cell cycle activation, PI3K/Akt/mTOR, and p53 [25]. Association with these pathways suggests that *MRPS12* may be involved in limiting apoptosis in cancer cells, which may explain the increased tumour recurrence in breast and ovarian cancers.

In terms of therapeutics, *MRPS12* has been associated with tamoxifen resistance in breast cancer [24], where combination therapies including tigecycline inhibited proliferation and enabled selective toxicity in cancer cells [33, 34]. In ovarian cancer, tigecycline improves response to chemotherapy in chemo-resistant and metastatic cells, and has minimal effect on normal ovarian cells, which can be attributed to a greater reliance on oxidative phosphorylation in these cells [35]. Interestingly, in the same study, tigecycline was found to suppress a range of signalling pathways, including mTOR signalling, and the cell cycle [35], which were upregulated in response to increased *MRPS12* expression in another study [25]. Further investigation into the effects of tigecycline and other tetracycline analogues on MRPS12 function may provide insight into this potential relationship.

#### *MRPS16*

Although the literature supporting a role for *MRPS16* is limited, there is evidence that this MRP may also prove to be a useful biomarker. In high-risk ovarian and lung cancers, the use of *MRPS16* as a biomarker of cancer progression has been demonstrated [26, 27]. Xu *et al.* (2021) examined the expression of six MRPs in a variety of ovarian cancer datasets, including TCGA, GTEx (Genotype-Expression Tissue Portal), and Oncomine, and found a strong positive correlation between increased *MRPS16* and poor survival in ovarian cancer [26]. Notably, *MRPS16* was upregulated in response to HE4 (human epididymis protein 4), an established ovarian cancer biomarker associated with metastasis [26]. *MRPS16* was among 40 mitochondrial-associated genes previously found to have genomic copy number variations in lung adenocarcinoma (LUAD) and lung squamous cell carcinoma (LUSC) [27]. Interestingly, *MRPS16* expression was consistently upregulated across both LUAD (n_cases_ = 533; n_controls_ = 59) and LUSC (n_cases_ = 502; n_controls_ = 49) TCGA samples, with significant differences between LUAD subtypes [27]. It is well documented that LUAD subtypes are differentiated by expression signatures and clinical outcomes [17]. This study by Hertweck *et al.* (2023) identified significant upregulation of *MRPS16* in the proximal-proliferative subtype compared to both the proximal-inflammatory and terminal respiratory unit groups [27]. Given the features of these subtypes, this suggests that *MRPS16* may contribute to decreased infiltration and increased proliferation in LUAD.

#### *MRPS18B*

One of the most interesting groups of MRPSs is the MRPS18 protein family which is comprised of three isoforms (MRPS18A, MRPS18B, MRPS18C) that bind to different sites of the mitochondrial ribosome [36]. MRPS18A is upregulated in non-small cell lung cancer (NSCLC) subgroups, while upregulation of MRPS18C is associated with poor prognosis in breast cancer [27, 31] (see Supplementary Material). Comparatively, MRPS18B has been associated with more than one cancer type. An increase in *MRPS18B* expression is also associated with EMT and metastatic capacity in endometrial and prostate cancer [28, 29]. Two studies by the same group used patient endometrium and prostate samples, as well as cell line and animal models, to study the relationship between *MRPS18B* and metastatic capacity [28, 29]. In endometrial cancer, they found that expression of MRPS18B was positively correlated with free E2F1 in 84 patient samples, with expression of both proteins significantly increased compared to normal and hyperplasia samples [28]* [E2F1 is a potential driver of metastasis and is highly expressed in late-stage endometrial tumours *[37]*].* Additionally, morphological changes in endometrial cancer cells expressing high levels of MRPS18B suggested progression of EMT, which was not observed in cells with lower MRPS18B expression [28]. In mouse endometrial cancer xenografts, MRPS18B overexpressing cells produced larger and more vascularised tumours, suggesting high expression increases proliferation and tumour aggressiveness *in vivo* [28]. Whereas, using a prostate cancer zebrafish xenograft model, experimental fish developed smaller tumours that migrated faster compared to those microinjected with low *MRPS18B* expressing cells [29]. The same study also found that increased expression of *MRPS18B* improved the migratory ability of prostate cancer cells through the induction of EMT, via chemokine signalling (CXCL12-CXCR4), and increased EMT transcription factor (TWIST2) expression [29]. Increased MRPS18B was correlated with disease stage, which suggests that EMT-induced migration increases metastasis [29]. In breast cancer, pathway enrichment analysis found that MRPS18B had roles in cancer-associated pathways including PIP3/AKT, oestrogen signalling, cell cycle, and circadian rhythm [30], however functional studies are needed to understand how MRPS18B impacts these pathways. Given the clear evidence that *MRPS18B* plays a crucial role in EMT progression and the development of metastatic capacity in multiple cancers, there is a clear need for further mechanistic studies manipulating *MRPS18B* expression to test its utility as a therapeutic target. The potential use as a biomarker of advanced disease requires larger cohort studies to understand the extent to which *MRPS18B* contributes to cancer progression, more broadly.

#### *MRPS31*

In ovarian and breast cancer, MRPS31 is known to interact with metastasis-associated proteins [26, 30]. Specifically, MRPS31 expression is associated with the established ovarian cancer biomarker, HE4 [26] and increased expression is associated with poor survival and poor progression free survival in ovarian cancer [26]. Which together suggests that it plays a role in ovarian tumour recurrence. In breast cancer, *MRPS31* is overexpressed in metastatic cell lines (MDA-MB231, MDA-MB468), and is known to interact with ACADSB [30] *[ACADSB is involved in fatty acid oxidation and has been associated with EMT and progression in a range of cancers, including breast cancer *[38–41]*]*. In triple negative breast cancer, MRPS31 also interacts with CES1 and NPAS2, both of which are involved in pathways that have been implicated in the progression and metastasis of a variety of cancers [30]. Additionally, there are multiple reports that *MRPS31* is associated with a range of other pathways associated with breast cancer progression, including PIP3/AKT, hedgehog signalling, and wnt signalling [30, 42–44].

#### *MRPS Summary*

In summary, over half of all MRPSs have been associated with metastatic capacity. Of the five that have been associated with multiple cancer types, *MRPS23* presents as an interesting candidate for further study. Given the broad foundation of work that has been performed across breast, cervical and hepatocellular carcinoma, as well as an association with numerous metastatic traits, this gene and protein clearly plays a role in metastatic capacity. Furthermore, there is compelling emerging evidence that increased *MRPS12, MRPS16* and *MRPS18B* expression and association with metastatic capacity are observed across five different cancer types, collectively. Therefore, it is apparent that MRPSs contribute to disease aggressiveness across the cancer spectrum.

### Large mitochondrial ribosomal proteins

Of the 52 proteins encoded by the large MRP genes, almost one quarter have been implicated in metastasis of more than one cancer type (Table 2) and the same proportion of MRPLs have been associated with metastasis of a single cancer type (see Supplementary Material). One of the most promising prognostic targets of cancer metastasis is MRPL4, with studies consistently demonstrating that increased expression is associated with a higher risk of metastasis in both breast and prostate cancer [24, 45, 46].

**Table 2:** Overview of the large mitochondrial ribosomal proteins associated with metastasis in more than one cancer type

*MRPL*	Associated Cancer(s)	Associated Protein(s) or Gene(s)	Associated Pathway(s)	Association of Metastatic Traits with Patterns of Expression
*MRPL1*	Breast [31], Lung [47] (LCLC), Colorectal [48]	SLC25A10 [48], Metastasis inhibition network (MRPL19, MRPL20, MRPL37, MRPL38, MRPL39, MRPL50, ICT1) [48]		▲ Poor Prognosis [31], ▲/▼ Risk of metastasis in lung cancer [47], ▼ Risk of metastasis in colorectal cancer [48]
*MRPL4*	Breast [24, 46] (ER + [46]), Prostate [45]	Part of a prognostic nine gene signature (*MRPL3, MRPL13, MRPL15, MRPL17, MRPL18, MRPL24, MRPL46, MRPL48*) [46]	RNA/mRNA binding [45], Ribosome signalling [45]	▲ Recurrence [24, 46], ▲ Risk of metastasis [45], ▲ Poor survival [45], ▲ Distant metastasis [46]
*MRPL9*	Lung [49], Hepatocellular [50]	MYC [49], ZEB1 [49], E-cadherin [49], Part of a prognostic two gene signature (*SMG5*) [50]	c-MYC signalling [49], Cell cycle [50], Mismatch repair signalling [50], Spliceosome signalling [50], Immune infiltration [50]	▼ Poor sphere formation [49], ▲ Risk of metastasis [49], ▲ Poor survival [49, 50], ▲ Recurrence [49, 50],▼ Poor proliferation [49, 50], ▼ Poor migration [49, 50], ▲ Drug resistance [50],
*MRPL10*	Ovarian [26], Lung [27] (LUAD; Proximal-proliferative subtype & LUSC; Classical subtype)	HE4 [26]		▲ Progression free survival [26], ▲ Poor survival [27]
*MRPL12*	Lung [51] (LUAD), Breast [52]		Immune infiltration [51]	▲ Poor prognosis [51], ▼ Poor proliferation [51], ▼ Poor invasion [51], ▼ Poor migration [52, 51], ▲ Poor survival [52],▼ Poor cell viability [52]
*MRPL13*	Breast [24, 31, 46, 52-55] (Triple-negative breast cancer [54, 55]), Lung (NSCLC [56], LUAD [57])	Part of multiple prognostic gene signatures [46], *VEGFA* [53], *MMP-2* [53], *MMP-9* [53], MYC [55, 56], Bcl-2 [56]	PI3K/AKT/mTOR [55, 56], Cell cycle [55–57], Immune infiltration [53, 57]	▲ Recurrence [24, 46, 54, 52, 55, 57], ▲ Poor Prognosis [31], ▲ Distant metastasis [46], ▼ Poor cell viability [52], ▼ Poor migration [52, 55, 57], ▲ Advanced stage [53, 55], ▲ Poor survival [53–57], ▲ Risk of metastasis [53, 55, 57], ▲ Proliferation [55–57], ▲ EMT [55, 57], ▲ Invasion [53, 57], ▲ Mutation burden [54], ▼ Hypoxia [57], ▼ Inflammation [57]
*MRPL15*	Breast [24, 46] (ER + [46]), Ovarian [26]	HE4 [26], MTORC1 [26], MYC [26], P27 [26], Part of multiple prognostic gene signatures [46]	Cell cycle [26]	▲ Drug resistance [24], ▲ Recurrence [24, 46], ▲ Poor progression free survival [26], ▲ Advanced stage [26], ▲ Risk of metastasis [26], ▲ Poor survival [26], ▲ Distant metastasis [46],
*MRPL19*	Colorectal [48], Lung [58] (LUAD)	MRPL1 [48], SLC25A10 [48], Metastasis inhibition network (MRPL20, MRPL37, MRPL38, MRPL39, MRPL50, ICT1) [48]	Proliferative signalling pathways [58], Cell cycle [58], Immune infiltration [58]	▼ Risk of metastasis in colorectal cancer [48], ▲ Risk of metastasis in LUAD [58], ▲ Invasion [58], ▲ Migration [58], ▲ Proliferation [58], ▲ Poor Survival [58], ▲ High grade [58]
*MRPL20*	Colorectal [48], Prostate [59]	MRPL1 [48], SLC25A10 [48], Metastasis inhibition network (MRPL19, MRPL37, MRPL38, MRPL39, MRPL50, ICT1) [48]	Hormone independence [59]	▼ Risk of metastasis [48], ▲ Androgen independence [59]
*MRPL35*	Lung (NSCLC) [60, 61], Colorectal [62], Gastric [63, 64]	*CDK1* [61], *BIRC5* [61], *CHEK1* [61], *STMN1* [61], *MCM2* [61], *SLC7A5* [60], *USP39* [60], PICK1 [63], BCL-XL [63], AGR2 [63], COPS5 [64], p53 [64]	p53 [61], Cell cycle [61, 62], Apoptosis [61, 64], Autophagy [62]	▲ Poor prognosis [60], ▲ Increased tumour size [60], ▼ Poor invasion [60], ▼ Low glutamine metabolism [60], ▼ Poor viability [60], ▲ Poor survival [60, 62, 63], ▼ Apoptosis [60, 62, 63], ▼ Poor proliferation [60–63], ▲ Metastasis [60, 63], ▲ Advanced stage [60, 63], ▼ Slowed tumour progression [61], ▼ Poor colony formation [62], ▼ DNA damage [62], ▼ Increased ROS [62], ▼ Poor tumour formation [63]
*MRPL36*	Ovarian [26], Breast [46, 65] (ER-/Basal [46])	HE4 [26], Part of a prognostic six gene signature (*MRPL13, MRPL22, MRPL41, MRPL42, MRPL54*) [46], Part of a prognostic four gene signature (*FEZ1, BMF, AFG1L*) [65]		▲ Poor progression free survival [26], ▲ Poor survival [26, 65], ▲ Risk of metastasis [26, 65], ▲ Distant metastasis [46], ▲ Mutation burden [65], ▼ Drug resistance [65], ▲ Advanced stage [65]
*MRPL37*	Lung [27] (LUAD; Terminal respiratory unit), Colorectal [48]	MRPL1[48], SLC25A10 [48], Metastasis inhibition network (MRPL19, MRPL20, MRPL38, MRPL39, MRPL50, ICT1) [48]		▼ Favourable prognosis [27], ▼ Risk of metastasis [48]
*MRPL38*	Lung [47] (LCLC), Colorectal [48]**,** Ovarian [66]	MRPL1 [48], SLC25A10 [48], Metastasis inhibition network (MRPL19, MRPL20, MRPL37, MRPL39, MRPL50, ICT1) [48]		▼ Risk of metastasis in colorectal and lung cancer [47, 48], ▲ Risk of metastasis in ovarian cancer [66], ▲ Migration [66]
*MRPL39*	Ovarian [26], Lung [47] (LCLC), Colorectal [48]	HE4 [26], TRAP1 [47], MRPL1 [48], SLC25A10 [48], Metastasis inhibition network (MRPL19, MRPL20, MRPL37, MRPL38, MRPL50, ICT1) [48]		▲ Poor survival [26], ▲ Advanced stage [26], ▼ Risk of metastasis [47, 48]
*MRPL42*	Breast [24, 46] (ER-/Basal [46]), Lung [67] (LUAD)	Part of a prognostic six gene signature (*MRPL13, MRPL22, MRPL36, MRPL41, MRPL54*) [46], YY1 [67], Vimentin [67],	Cell cycle [67]	▲ Recurrence [24], ▲ Distant metastasis [46], ▲ Invasion [67], ▲ Migration [67], ▲ Proliferation [67], ▲ Risk of metastasis [67]
*MRPL44*	Breast [24], Thyroid [68]		Oxidative Phosphorylation [68]	▲ Recurrence [24], ▲ Risk of metastasis [68]
*MRPL49*	Lung [27] (LUAD & LUSC), Breast [69]			▲ Advanced stage [27], ▼ Invasion [69]
*MRPL54*	Hepatocellular [70, 71], Breast [46] (ER-/Basal)	Part of a prognostic six gene signature (*MRPL13, MRPL22, MRPL36, MRPL41, MRPL42*) [46], Part of a prognostic six gene signature (*CNOT6, UPF3B, ZC3H13, IFIT5, PPARGC1A*) [70], Part of a prognostic four gene risk model (*EZH2, PPARGC1A, EIF2AK4*) [71]		▼Poor Survival [70, 71], ▲ Distant metastasis [46]

▲Increased expression of MRP is associated with the trait; ▼ Decreased expression of MRP is associated with the trait. Abbreviations: LUAD, lung adenocarcinoma; LUSC, lung squamous cell carcinoma; ER, estrogen receptor; NSCLC, non-small cell lung cancer; LCLC, large cell lung cancer; EMT, epithelial to mesenchymal transition

An overview of the large mitochondrial ribosomal proteins associated with metastasis in only one cancer type can be found in Supplementary Table 1

#### *MRPL4*

In prostate cancer, comparison of expression between benign prostatic hyperplasia and low- and high-risk primary prostate cancer samples, revealed 56 proteins, including MRPL4, which were highly expressed in the high-risk samples compared to the other two groups [45]. *MRPL4* gene expression in high-risk samples was also more than double that of low-risk and benign prostatic hyperplasia samples [45]. Interestingly, expression of MRPL4 was found to have a greater association with high-risk samples than AMACR, an established biomarker of prostate cancer [72], with MRPL4 overexpression also associated with increased mortality and poorer survival [45, 72]. Similarly, Sotgia *et al.* (2017) observed, through an informatics-based approach, that *MRPL4* expression was associated with tumour recurrence and hormone resistance in breast cancer, further suggesting a role in aggressive cancer phenotypes [24]. Microarray data from 3,951 breast cancer tumours additionally revealed that increased expression of *MRPL4* was associated with distant metastasis in estrogen-receptor positive patients [46].

#### *MRPL13*

In addition to MRPL4, MRPL13 has been widely studied across breast and lung cancer and holds promise as a putative metastatic prognostic target. *MRPL13* was first identified as a potential prognostic biomarker in breast cancer [24]. Using an informatics-based approach, one study observed that *MRPL13* expression was associated with breast cancer tumour recurrence and hormone resistance across several Gene Expression Omnibus (GEO) datasets (n = 3,455) [24]. Analyses of breast cancer TCGA data showed high *MRPL13* expression correlated with poor prognosis, as well as advanced tumour stage, increased immune infiltration and metastasis [53]. Analysis of microarray data from 3,951 breast cancer tumours also found that recurrence and distant metastasis were associated with increased *MRPL13* [46]. These findings indicate that increased *MRPL13* expression likely contributes to aggressive cancer phenotypes with potential clinical applications in predicting response to therapy [31, 46, 53, 54]. Furthermore, *in vitro* studies have found that downregulation of *MRPL13* inhibited migration and decreased cell viability in bone and lung metastasised breast cancer cell lines [52]. Another study found that *MRPL13* knockdown inhibits invasion in both non-invasive (MCF-7, T47D) and invasive (MDA-MB-231) breast cancer cells [53, 55], partly through diminished EMT processes [55], which suggests that *MRPL13* expression may contribute to the acquisition of metastatic traits in breast cancer. Upregulation of *MRPL13* is also associated with lung cancer [54, 56, 57]. Specifically, a TCGA Pan-Cancer Atlas analysis revealed that *MRPL13* is significantly upregulated in LUAD patient samples [54]. Likewise, an additional study found increased *MRPL13* mRNA and protein expression in NSCLC tumours compared to normal tissue, with the highest expression observed in a metastatic NSCLC cell line (H1299) [56]. This study also found an association between *MRPL13* expression and MYC, PI3K/AKT/mTOR, metabolism, cell cycle pathways, and increased proliferation, suggesting links to cancer progression and potentially EMT promotion [56]. This was additionally supported by analysis of the TCGA LUAD dataset that found *MRPL13* expression was associated with immune infiltration, metastasis, and EMT, with gene knockdown *in vitro* decreasing cell survival and metastasis, and increasing apoptosis [57]. Furthermore, Zhong *et al.* (2023) found that by employing *MRPL13* as a diagnostic biomarker, the accuracy of diagnostic predictions across multiple cancer types, including LUAD, were improved [57], which is a promising area of future research.

Recently, Li *et al.* (2023) suggested the use of synthetic nucleic acid drugs to disrupt the production of the MRPL13 protein (as well as MRPL9; see relevant section below) [49]. The use of synthetic nucleic acids as therapeutics is relatively new but provides a compelling opportunity to directly target genes that promote metastatic capacity in cancers. The therapy selectively targets tissues or cells with oligonucleotides that only bind to target RNA, which modulates function or promotes degradation, thus allowing the synthesis of specific proteins to be inhibited [73]. Several antisense oligonucleotide therapies have been approved by the Food and Drug Administration (FDA) and the European Medicines Agency (EMA), although none are currently approved for cancer treatment [74]. Therefore, repurposing them to target MRPs, such as *MRPL13* and *MRPL9* in aggressive lung cancer is a promising area of future research. We suggest that studies should firstly aim to assess the underlying mechanisms by which these *MRPLs* promote metastasis using *in vitro* models. Such therapies can then be tested in these models to determine whether they specifically target *MRPL9* and *MRPL13*, and whether they adequately suppress the metastatic phenotype, before they can enter clinical trials.

#### *MRPL36*

MRPL36 is another example of a promising risk predictor, particularly in relation to breast and ovarian cancer metastasis [26, 46, 65]. Recently, *MRPL36* was included in a four gene breast cancer risk prediction model and, of the four genes, was determined to be the most predictive of risk [65]. This risk score was capable of predicting not only survival of breast cancer patients, but also tumour stage, including metastasis [65]. In a second study, increased expression of *MRPL36* was associated with distant metastasis in estrogen-receptor negative breast cancer [46]. Similarly, Xu *et al.* (2021) revealed an apparent association between high *MRPL36* expression and upregulation of HE4 in ovarian cancer [26]. Collectively, increased expression of *MRPL36* has been linked to a wide variety of metastatic traits (Table 2), thus further evidence of its importance in cancer metastasis across a broader range of cancer types would be of value.

#### *MRPL35*

As for the most promising MRPL therapeutic targets, studies have targeted MRPL35 and MRPL42 with 18β-glycyrrhetinic acid and a YYI inhibitor, respectively. Increased *MRPL35* expression is associated with poor survival outcomes across NSCLC, colorectal, and gastric cancers [60–64]. In NSCLC, high expression is correlated with advanced stage and metastasis, with knockdown inhibiting proliferation, invasion, and glutamine metabolism [60]. The authors found that *MRPL35* knockdown caused downregulation of SLC7A5 [60], which is a lung cancer-specific prognostic biomarker [75]. Additionally, knockdown promoted apoptosis and impeded proliferation, likely through the modulation of several cell regulatory proteins (CDK1, BIRC5, CHEK1, STMN1 and MCM2) and the activation of the p53 signalling pathway [61]. In colorectal cancer, downregulation of *MPRL35* increased reactive oxygen species production leading to DNA damage, cell cycle arrest, decreased mitochondrial membrane potential, and ultimately, apoptosis [62]. As a result, knockdown of *MRPL35* inhibited proliferation and colony formation, *in vitro* and *in vivo*, using a xenograft mouse model [62]. In gastric cancer, increased expression of *MRPL35* is associated with metastasis and advanced stage, with *in vivo* knockdown inhibiting tumour formation and promoting apoptosis [63]. Later investigation by the same authors found that treatment of gastric cancer cell lines (BGC-823, MGC80-3) with 18β-glycyrrhetinic acid, a promising anti-inflammatory and antioxidant agent, inhibited *MRPL35* expression and induced cancer cell apoptosis and cell cycle arrest [64]. Together, these findings suggest *MRPL35* may have an application as a therapeutic target across a range of cancers, however further investigations beyond gastric cancer are required.

#### *MRPL42*

In LUAD, MRPL42 is known to support metastatic properties, and is crucial for tumour growth and development [67]. Jiang and colleagues (2021) demonstrated that knockdown of *MRPL42* decreased migration, invasion, and proliferation in LUAD cells of varying metastatic capacities [67]. Additionally, expression of *MRPL42* is correlated with the presence of lymph node metastases and tumour size in patient samples [67]. This study found that the transcription factor, YY1, was likely responsible for increased *MRPL42* expression, with knockdown of YY1 associated with decreased *MRPL42* expression [67]. Consistent with these findings, high levels of *MRPL42* are associated with tumour recurrence in breast cancer patients and are predictive of distant metastasis in estrogen-receptor negative basal breast cancer [24, 46], as shown in Table 2. We suggest that if MRPL42 expression could be therapeutically targeted through the inhibition of YY1 [67], then the size of MRPL42-overexpressing LUAD tumours could be reduced and the presence of lymph node metastases significantly diminished [47]. The same may be possible in breast cancer, where MRPL42 also appears to play a significant role in aggressive disease.

#### *MRPL1*

Although the literature supporting a role for *MRPL1* is limited, there is evidence that this MRP may also prove to be a useful biomarker. *MRPL1* has been implicated in the progression of breast, colorectal, and large cell lung cancer (LCLC) [31, 47, 48]. In breast cancer, through the analysis of 1,056 TCGA tumours, increased expression of *MRPL1* was associated with poor prognosis and over 50% increased risk of mortality [31]. Conversely, a broad investigation into metastasis-associated genes in colorectal cancer proposed that MRPL1 was linked to decreased metastatic risk via SLC25A10 [48] [*SLC25A10 is a mitochondrial translocation protein that is often overexpressed in tumour cells, and is associated with rapid proliferation* [76]]. This suggests that MRPL1 may play a role in excessive proliferation in primary cancers, in turn suppressing the migratory capacity of metastatic cells. Analysis of mitochondrial protein profiles from LCLC cell lines with varying metastatic potential, revealed 64 differentially expressed proteins, including MRPL1 [47]. Though, contrary to the findings in breast and colorectal cancer, the role of MRPL1 in LCLC remains inconclusive.

#### *MRPL9*

In lung cancer and hepatocellular carcinoma, *MRPL9* overexpression is associated with poor survival and recurrence, and knockdown in an *in vitro* cell model inhibited pro-metastatic capabilities such as migration, spheroid formation, and proliferation [49, 50]. *MRPL9* was found to modulate c-MYC transcription, which alters ZEB1 expression to regulate the expression of e-cadherin in lung cancer [49]. High *MRPL9* expression is known to be associated with decreased e-cadherin expression, which is essential for cancer-specific EMT [77]. In hepatocellular carcinoma, *MRPL9* was used in a two gene prognostic model that could predict prognosis, immune infiltration, and chemoresistance [50]. This model was effective in identifying patients suitable for immunotherapy and those who were likely to have better survival outcomes post chemotherapy [50]. *MRPL9* was found to be associated with chemoresistance pathways in hepatocellular carcinoma, including cell cycle, mismatch repair, and spliceosome signalling [50]. Together, these studies suggest *MRPL9* may play a key role in aggressive cancer phenotypes and could be a viable therapeutic target.

#### *MRPL10*

*MRPL10* has been linked to lower tumour recurrence in ovarian cancer, however it has also been associated with aggressive molecular subtypes in LUAD and LUSC [26, 27]. In ovarian cancer, through gene expression analysis of tumours, significant upregulation of *MRPL10* in response to HE4 overexpression has been observed, although *MRPL10* overexpression was also correlated with progression free survival [26]. This suggests that increased expression of *MRPL10* may be a protective response to upregulation of HE4 in ovarian cancer. Whereas, high expression of *MRPL10* was observed to correlate with aggressive molecular subtypes of both LUAD and LUSC, through an analysis of TCGA data by Hertweck and colleagues (2023) [27]. In LUAD, *MRPL10* expression was higher in the proximal-proliferative subtype, which is often associated with poorer outcomes [27, 78, 79]. Similarly, in LUSC, higher *MRPL10* expression was observed in the classical subtype [27], which is often associated with drug resistance [79, 80]. Thus, these studies suggest that the role of *MRPL10* may vary between cancer types.

#### *MRPL12*

Knockdown of *MRPL12* is associated with decreased metastatic capacity, such as proliferation, migration and cell viability, in aggressive lung and breast cancer cell lines [51, 52]. This response to loss of *MRPL12,* in both LUAD and breast cancer, suggest that it plays a key role in promoting metastatic behaviour and tumour growth. Further supporting this, the same study found that high expression of *MRPL12* was associated with worse prognosis, immune infiltration and poor survival in LUAD tumours [51, 52]. Due to *MRPL12’s* association with prognostic factors and metastatic capacity in breast cancer, Liu *et al.* incorporated MRPL12 into a three-protein prognosis prediction signature, alongside MRPL13 and POP1 [52]. Combined with age and stage data, this signature was able to determine short-term survival for breast cancer patients and highlighted these genes as possible therapeutic targets [52].

#### *MRPL15*

*MRPL15* has shown prognostic potential in both breast and ovarian cancers, with expression correlating with recurrence and risk of metastatic disease [24, 26, 46]. Among 12 MRPs examined in breast cancer, *MRPL15* had the best prognostic value [24]. Additionally, *MRPL15* was incorporated into a four gene signature, where patients with a high signature expression were over five times more likely to experience tumour recurrence and over three times more likely to develop metastasis, compared to those with a low signature expression [24]. Similarly, Xu and colleagues (2021) demonstrated the potential use of *MRPL15* as an ovarian cancer prognostic biomarker and therapeutic target [26]. *MRPL15* was consistently highly expressed in ovarian tumours compared to normal tissue; significantly associated with late stage disease, and was associated with poor survival when compared to patients with low *MRPL15* expression [26]. Additionally, *MRPL15* is known to correlate with HE4, and their interaction is thought to promote oncogenesis via increased metastatic capacity and drug resistance [26].

#### *MRPL19*

In LUAD, *MRPL19* has been suggested as a potential biomarker as it is associated with markers of cancer progression and metastasis [58]. Upregulation of *MRPL19* in LUAD samples is associated with poor prognosis, including increased differentiation, tumour stage, and metastasis [58]. Notably, knockdown of *MRPL19* in LUAD cells inhibited cell growth, migration, and invasion, further supporting links to metastatic capacity [58]. This study additionally identified correlations between *MRPL19* expression and proliferative signalling pathways, as well as cell cycle, adhesion molecules, and immune infiltration pathways [58]. Considered together, these findings provide a very compelling connection between *MRPL19* and metastasis in LUAD cells. Conversely, a broad investigation into metastasis-associated genes in colorectal cancer proposed that MRPL19 was associated with decreased metastasis risk through SLC25A10 and MRPL1 [48]. This suggests that the role of *MRPL19* may be multifaceted, with different functions across a range of cancers.

#### *MRPL20*

Differential expression of *MRPL20* has been observed in prostate and colorectal cancers [48, 59]. In prostate cancer, comparison of gene expression profiles between newly-diagnosed, androgen-dependent and androgen-independent primary tumours from patients with metastatic disease [59], revealed higher *MRPL20* expression in androgen-independent samples, suggesting a possible role in aggressive prostate cancer [59]. Conversely, *MRPL20*, was found to limit metastasis in colorectal cancer [48].

#### *MRPL37*

In LUAD, *MRPL37* appears to contribute to more aggressive tumour subtypes, while it has been indicated as part of a metastasis inhibition network in colorectal cancer [27, 48]. Upregulation of *MPRL37* has been observed in both LUAD and LUSC cases compared to unaffected controls [27] and downregulation of *MRPL37* was significantly associated with the terminal respiratory unit subtype in LUAD [27], which has a favourable prognosis. This suggests that upregulation of *MRPL37* may contribute to aggressive lung tumour subtypes [17]. Contrarily, *MRPL37* was associated with metastasis inhibition in colorectal cancer, with interactions with SLC25A10 through MRPL1 also identified [48].

#### *MRPL38*

*MRPL38* is associated with fewer metastatic lesions in both lung and colorectal cancers [47, 48]. Analysis of mitochondrial protein profiles from LCLC cell lines, revealed that cells with high metastatic potential had lower MRPL38 expression compared to cells with low metastatic potential [47]. Likewise, in colorectal cancer, analysis of genes associated with metastasis found that *MRPL38* was associated with lower levels of metastasis through SLC25A10 [48]. Conversely, MRPL38 is associated with heightened metastatic capacity in ovarian cancer cells, where a more invasive cell line exhibited a three-fold increase in expression compared to a paired non-invasive cell line [66]. These findings suggest a more nuanced role for *MRPL38* in metastatic progression, which is dependent on tumour characteristics.

#### *MRPL39*

Studies in ovarian, lung, and colorectal cancers, provide some insight into the potential mechanisms of *MRPL39* in tumour progression and metastasis [26, 47, 48]. In ovarian cancer, *MRPL39* was among the top six MRPs whose expression correlated with tumorigenicity [26], including advanced stage and poor survival [26]. A study in LCLC cells by Liu* et al.* (2019), revealed 64 differentially expressed proteins, including MRPL39, when comparing cells with low and high metastatic potential [47]. Higher levels of MRPL39 were observed in cells with lower metastatic potential, suggesting a tumour suppressive role [47]. Additionally, this study proposed that MRPL39 functions in a similar manner to TRAP1, which is a regulator of mitochondrial respiration. High expression of TRAP1 in LUAD is known to increase proliferation but inhibit metastasis [47, 81]. Notably, in a colorectal cancer study, *MRPL39* was associated with reduced metastasis [47, 48, 81]. As previously mentioned, this study uncovered metastasis inhibiting protein–protein interactions, connecting MRPL39 to SLC25A10, through MRPL1 [48]. These findings indicate that *MRPL39* favours metastatic progression, particularly in ovarian cancer, but appears disadvantageous to metastasis in others, such as colorectal and lung cancers.

#### *MRPL44*

In thyroid cancer, patients with unaltered *MRPL44* expression between tumour and benign tissue were observed to have an increased risk of lymph-node metastasis compared to those with decreased expression in tumour tissue [68]. Additionally, low *MRPL44* expression correlated with a glycolytic metabolic phenotype and a lower risk of metastasis, while high expression indicated a combined oxidative phosphorylation and glycolysis phenotype [68]. The latter may confer a growth advantage thereby increasing metastatic potential [68]. *MRPL44* expression positively correlated with the expression of proteins involved in electron transport, mitochondrial metabolism, and apoptosis, providing a potential mechanism for metabolic control. Similarly, one study observed a correlation between high *MRPL44* expression and an increased risk of tumour recurrence in breast cancer [24]. These studies indicate a role for *MRPL44* in the promotion of metastasis, however investigation into the effects of altered expression on metabolism and hormone resistance may elucidate its exact role.

#### *MRPL49*

Altered *MRPL49* expression has been observed in breast and lung cancer, with a potential link to metastasis [27, 69]. In breast cancer, MRPL49 expression appears to support tumour development, but reduces metastatic capacity [69]. Specifically, a study observed upregulation of MRPL49 in non-invasive breast cancer cells, and downregulation in invasive cells compared to normal breast epithelial cells [69]. This suggests that MRPL49 modulates invasive capacity, perhaps through control of mitochondrial metabolism. Consistent with the Warburg effect, primary non-invasive cells may utilise oxidative phosphorylation to increase proliferation, with a switch towards glycolysis in invasive cells [82]. Interestingly, enrichment of the glycolysis pathway correlated with EMT in primary breast cancer, which could suggest that downregulation of MRPL49 in invasive cells is linked to EMT and metastatic progression [69, 83]. However, an alternate pattern of *MRPL49* expression was observed in a study utilising TCGA lung cancer data [27]. Specifically, *MRPL49* expression was increased in late-stage LUAD and LUSC tumours compared to normal lung samples [27]. These studies may indicate that cancer cells require varying metabolic needs during different stages of tumour progression and further research is needed to elicit the potential role of *MRPL49* in mitochondrial metabolism and cancer.

#### *MRPL54*

*MRPL54* expression has been used to predict outcomes in hepatocellular carcinoma and basal breast cancer [46, 70, 71]. *MRPL54* was used in two separate survival risk prediction gene signatures in hepatocellular carcinoma patients [70, 71]. One of these studies additionally found that *MRPL54* overexpression was a protective factor associated with better outcomes, with the authors suggesting it may function as a tumour suppressor gene [70]. In oestrogen receptor-negative basal breast cancer, *MRPL54* was part of a six gene signature that could effectively predict distant metastasis, recurrence, and overall survival [46]. Further investigation to understand the function and mechanism by which *MRPL54* may contribute or prevent cancer progression is warranted.

#### *MRPL Summary*

In summary, 12 MRPLs have been associated with lung cancer, 11 with breast, seven with colorectal, six with ovarian, two with prostate, two with liver (hepatocellular carcinoma), and only one with thyroid and one with gastric. Many of the MRPLs discussed throughout this review have conflicting evidence in the literature between cancer types. For example, in ovarian cancer, increased *MRPL10* expression may have a protective response [26], whereas in lung cancer, high expression is associated with poorer outcomes and drug resistance [27, 78, 79]. Therefore, further investigation into the pathways effected by *MRPL10* expression would assist in clarifying the protein’s role in cancer progression across the cancer spectrum. Likewise, for the other MRPLs that appear to have different functions between cancer types (e.g. *MRPL19, MRPL37* and *MRPL49*). Studies suggest that differences in tumour microenvironment, metabolic phenotype, or cell type may alter their role in cancer progression. Conversely, MRPL genes with consistently established associations between expression and metastasis-related traits across a variety of cancer types (e.g. *MRPL4, MRPL35* and *MRPL42*), are putative prognostic and therapeutic targets as they would have broad applicability across the cancer spectrum, thus directly benefit more patients. Although there are a number of MRPLs (and MRPSs) that have conflicting findings between cancers or have only been associated with one cancer type (as shown in the Supplementary Material), where strong evidence of therapeutic value has been demonstrated, these putative therapeutic targets remain worthy of investigation given the dearth of targeted options currently available for advanced disease.

## Therapeutically targeting mitochondrial-associated ribosomal proteins in metastatic cancer

There is currently no cure for metastatic cancer and there are very few treatment strategies available for patients with advanced high-grade disease. Therefore, there is an urgent need to find a cure for this devastating condition. We believe that therapeutically targeting markers of metastasis, such as MRPs, is crucial to preventing disease progression. Although the use of MRPs as therapeutic targets in metastatic cancer has shown great promise, there are currently very few studies in the literature. This review has identified *MRPL4, MRPL13* and *MRPL36* as potential prognostic targets, and *MRPS12, MRPL35* and *MRPL42* as putative therapeutic targets from the current literature. Where there are direct links with specific MRPs with therapeutic options in the literature, we have provided further details in the previous sections, but there are additional promising avenues for targeting MRPs we would like to highlight.

Broad approaches to targeting MRP function, such as mitochondrial ribosome inhibition or disturbing MRP synthesis, have been shown to prevent drug resistance and metastasis in a variety of cancers [84, 85]. Mitoriboscins are a recently identified group of mitochondrial-related antibiotics that are capable of binding to, and inhibiting, the mitochondrial ribosome [84]. These drugs have been shown to preferentially target cancer stem cells and groups of cancer cells in breast cancer cell models, effectively inhibiting oxidative phosphorylation, cell viability, migration, and cancer stem cell propagation, while showing no effect on normal fibroblasts [84]. Another study showed that Mitoriboscins had little effect on tumour growth but did inhibit metastasis, with very low embryo toxicity [46], thus proving its potential as a metastatic cancer therapeutic option. Furthermore, MRP synthesis can be targeted by the HSP70 inhibitor, JG-98, which can disrupt MRP stability through misfolding in castration-resistant prostate cancer [85]. The study observed decreased expression of all five MRPs tested (MRPS27, MRPS23, MRPS17, MRPL44, MRPL19), reduced mitochondrial respiration, and found that JG-98 sensitised castration-resistant prostate cancer cells to androgen-deprivation therapy [85]. Whilst this study highlights how MRP synthesis can be effectively inhibited in prostate cancer, we suggest that perhaps further assessment of this mechanism in a more relevant cancer cell model would enable the efficacy of this treatment to be teased apart. Notably, most of the studies highlighted in this review were undertaken in lung, breast and ovarian cancer, which may provide a starting point for further studies examining the effect of HSP70 inhibitors.

Moreover, repurposing existing FDA approved therapies is a promising avenue for targeting MRPs in aggressive cancer. Repurposing FDA approved therapies has enabled the timely use of effective and well-studied therapeutics for a range of conditions, including cancer. For example, tetracycline analogues, such as doxycycline, COL-3 and tigecycline, are approved for use as broad-spectrum antibiotics to treat a wide variety of bacterial infections [86], however they have shown anti-tumour effects in several cancers in both *in vitro* studies and clinical trials [87–89]. These medications have also been shown to inhibit the process of mitochondrial translation, in which MRPs play a key role [87–89]. The possible effects of tetracycline analogues on MRP expression remains unclear and has not yet been studied, however, tigecycline is thought to inhibit the function of the mitochondrial ribosome, exhibiting selective inhibition of mitochondrial translation, biogenesis, and respiration to induce apoptosis in cancer cells [35, 90]. Given the known effect of tigecycline on *MRPS12* expression, we suggest that studies assessing the interplay between tumour recurrence, tamoxifen resistance and *MRPS12* expression is an ideal starting point. Additionally, pentamidine, a broad-spectrum antimicrobial drug used to treat several parasitic worms, protozoa, and fungi, has been found to inhibit prostate cancer progression *in vitro* by inducing mitochondrial DNA depletion and dysfunction [91]. Again, no study has assessed its effect on MRP expression and function, specifically, which is an obvious area of future research.

## Conclusion

In addition to their normal function, MRPs play diverse roles in regulating the survival, development, and progression of cancers, particularly those that are advanced and metastatic. However, our understanding of how MRPs mechanistically contribute to the growth and progression of metastatic cancer is very limited. The majority of studies suggest that MRPs play key roles in driving proliferation and migration, as well as EMT, and propose that MRPs may promote the acquisition of metastatic properties in cancer cells. This review has explored studies which have assessed the expression of MRPs in cell lines and patient samples from a range of cancer types, and in some cases, associations with clinical characteristics and patient outcomes. Due to their expression profile in cancer cells and the role they are known to play in advanced disease, MRPs could provide therapeutically exploitable opportunities in a variety of advanced cancers, however there is limited literature in the area. Numerous studies have found conflicting data across cancer types and perhaps a systematic approach to unravelling the role MRPs play in metastasis would be more beneficial than studies assessing MRPs in individual cancers. MRPs associated with more than one cancer type hold the greatest potential as prognostic and therapeutic targets of metastasis given their broad application across the cancer spectrum. The authors conclude that targeting the mitochondria is a burgeoning field of cancer research and further investigations into the role of MRPs in cancer progression is warranted.

## Supplementary information

Below is the link to the electronic supplementary material.

Supplementary file1 (DOCX 86 KB)

## Data Availability

No datasets were generated or analysed during the current study.
