# Supplementary material for: Mitochondrial ribosomal proteins in metastasis and their potential use as prognostic and therapeutic targets
Source: Cancer Metastasis Rev. 2024 Oct 1;43(4):1119–35. doi: 10.1007/s10555-024-10216-4 (PMC11554709; doi:10.1007/s10555-024-10216-4)
Supplement: Supplementary file 1 — Supplementary file1 (DOCX 86 KB) [file 10555_2024_10216_MOESM1_ESM.docx]

# Mitochondrial ribosomal proteins in metastasis and their potential use as prognostic and therapeutic targets

Jasmine M Bacon^1^, Johanna L Jones^1^, Guei-Sheung Liu^2,3,1^, Joanne L Dickinson^1^, Kelsie Raspin^1,^^

^1^ Menzies Institute for Medical Research, University of Tasmania, Hobart, Tasmania, Australia

^2^ Centre for Eye Research Australia, Royal Victorian Eye and Ear Hospital, East Melbourne, Victoria, Australia

^3^ Ophthalmology, Department of Surgery, University of Melbourne, East Melbourne, Victoria, Australia

^^^ To whom correspondence should be addressed. Tel +61 6226 4762; Email: [kelsie.raspin@utas.edu.au](mailto:kelsie.raspin@utas.edu.au); ORCID: 0000-0001-8463-6820

## Supplementary Table 1. Overview of mitochondrial ribosomal proteins associated with one type of metastatic cancer.

| *MRP* | Associated Cancer | Associated Protein(s) or Gene(s) | Associated Pathway(s) | Association of Metastatic Traits with Patterns of Expression |
| --- | --- | --- | --- | --- |
| Breast Cancer | | | | |
| *MRPS6* | Breast [1, 2] | AKAP10 [1], E-cadherin [1], Oncogenes (IGF1, CCND1, EZH2, MYC, GLI1) [1], Tumour suppressor genes (LMNA, TGFBR2) [1] |  | ▲ Proliferation [1], ▲ EMT [1], ▲ Oncogene expression [1], ▼ Tumour suppressor expression [1], ▲ Poor survival [1, 2], ▲ Poor prognosis [2] |
| *MRPS9* | Breast [3] | BCKDHB [3], NPAS2 [3], ROS1 [3] | ROS1 interaction [3], PIP3/AKT pathway [3], MAPK signalling [3], Apoptosis [3], Hedgehog signalling [3], Cell cycle [3] |  |
| *MRPS10* | Breast [3] | ACADSB [3], TDRKH [3] | BDNF signalling pathway [3], Fatty acid oxidation [3], Nonsense-Mediated Decay [3], p53 pathway [3] | ▲ Metastatic cell line [3], ▲ Fatty acid oxidation [3] |
| *MRPS11* | Breast [3, 4] | BCKDHB [3], FRAP1 [3], NPAS2 [3], ROS1 [3] | Oestrogen signalling pathway [3], NF-jB pathway [3], Wnt signalling [3] | ▲ Drug Resistance [4], ▲ Recurrence [4] |
| *MRPS18C* | Breast [2] | Part of a four gene nomogram, with age and stage, to predict survival (MRPL16, MRPL40, MRPS35) [2] |  | ▲ Poor prognosis [2], ▲ Poor survival [2] |
| *MRPS22* | Breast [3, 4] | p53 [3] | MAPK6/MAPK4 signalling [3], Hedgehog signalling pathway [3] | ▲ Recurrence [4] |
| *MRPS28* | Breast [3, 4] | p53 [3] |  | ▲ Recurrence [4] |
| *MRPS33* | Breast [3, 4] |  | PIP3/AKT pathway [3], MAPK signalling [3], Hedgehog signalling [3],  NF-jB pathway [3], Oncogene-induced senescence [3], Wnt signalling [3], Spliceosome [3] | ▲ Recurrence [4] |
| *MRPS35* | Breast [2] | Part of a four gene nomogram, with age and stage, to predict survival (MRPL16, MRPL40, MRPS18C) [2] |  | ▲ Poor prognosis [2], ▲ Poor survival [2] |
| *MRPL3* | Breast [4, 5] |  |  | ▲ Recurrence [4, 5], ▲ Distant metastasis [5] |
| *MRPL16* | Breast [2] | Part of a four gene nomogram, with age and stage, to predict survival (MRPL40, MRPS18C, MRPS35) [2] |  | ▼ Poor prognosis [2], ▼ Poor survival [2] |
| *MRPL17* | Breast [4, 5] (ER+ [5]) |  |  | ▲ Recurrence [4, 5], ▲ Distant metastasis [5] |
| *MRPL18* | Breast [4, 5] (ER+ [5]) |  |  | ▲ Drug Resistance [4], ▲ Recurrence [4, 5], ▲ Distant metastasis [5] |
| *MRPL22* | Breast [5] (ER-/Basal) |  |  | ▲ Distant metastasis [5] |
| *MRPL23* | Breast [4] |  |  | ▲ Recurrence [4] |
| *MRPL24* | Breast [4, 5] (ER+ [5]) |  |  | ▲ Recurrence [4, 5], ▲ Distant metastasis [5] |
| *MRPL40* | Breast [2] | Part of a four gene nomogram, with age and stage, to predict survival (*MRPL16, MRPS18C, MRPS35*) [2] |  | ▼ Poor prognosis [2], ▼ Poor survival [2] |
| *MRPL41* | Breast [5] (ER-/Basal) |  |  | ▲ Distant metastasis [5] |
| *MRPL46* | Breast [4, 5] (ER+ [5]) |  |  | ▲ Recurrence [4, 5], ▲ Distant metastasis [5] |
| *MRPL48* | Breast [4, 5] (ER+ [5]) |  |  | ▲ Recurrence [4, 5], ▲ Distant metastasis [5] |
| *MRPL52* | Breast [6] | HIF-1 [6], Snail [6], N-cadherin [6], Vimentin [6], E-cadherin [6],  ZO-1 [6], PINK1 [6], Parkin [6] | Hypoxic response [6], Mitophagy [6],  ROS homeostasis [6], ROS-Notch1-Snail signalling pathway [6] | ▲ Metastasis [6], ▼ Apoptosis [6], ▲ Migration [6], ▲ Invasion [6], ▲ EMT [6], ▲ Recurrence [6], ▼ ROS accumulation [6], ▲ Survival/malignancy in hypoxic environments [6] |
| Other Cancers | | | | |
| *MRPL50* | Colorectal [7] | MRPL1 [7], SLC25A10 [7], Metastasis inhibition network (MRPL19, MRPL20, MRPL37, MRPL38, MRPL39, ICT1) [7] |  | ▼ Risk of metastasis [7] |
| *MRPS17* | Gastric [8] | p53 [8], AKT [8] | PI3K/AKT [8], Cell adhesion [8], Extracellular matrix [8] | ▲ Invasion [8], ▲ Poor survival [8], ▼ Poor proliferation [8],  ▲ Risk of metastasis [8],  ▲ Poor prognosis [8], ▲ Advanced stage [8] |
| *MRPL59* | Hepatocellular [9] | miR-497-5p [9] | Cell cycle [9], ROS homeostasis [9], NFκB signalling pathway [9] | ▲ Poor prognosis [9], ▲ Poor survival [9], ▲ Recurrence [9], ▲ Inhibited apoptosis [9],  ▲ EMT [9], ▲ Metastasis [9], ▲ High grade [9], ▲ Late stage [9], ▲ Increased tumour size [9], ▲ ROS accumulation [9] |
| *MRPS18A* | Lung [10] (LUAD (Proximal-proliferative subtype)) |  |  | ▲ Poor survival [10] |
| *MRPL27* | Lung [11] (KRAS MT) |  |  | ▲ Poor survival [11], ▲ Poor prognosis [11] |
| *MRPL51* | Lung [12] (LUAD) | FOXM1 [12], N-cadherin [12], E-cadherin [12], Vimentin [12] | DNA repair [12], Cell cycle [12], | ▲ EMT [12], ▲ Invasion [12], ▲ Proliferation [12], ▲ Poor survival [12], ▲ Poor progression free survival [12] |
| *MRPS7* | Osteosarcoma [13] |  |  | ▲ Metastasis [13] |
| *MRPL57* | Prostate [14] |  | Hormone independence [14] | ▲ Androgen independence [14] |
| *MRPL14* | Thyroid [15] | EMT-related proteins [15], MTCOT1 [15] | ROS homeostasis [15] | ▲ Advanced stage [15], ▲ Metastasis [15], ▲ Proliferation [15], ▲ Migration [15], ▲ EMT [15], ▼ ROS accumulation [15] |
| ▲ Increased expression is associated with the trait; ▼ Decreased expression is associated with the trait. Abbreviations: LUAD, lung adenocarcinoma; ER, estrogen receptor; EMT, epithelial to mesenchymal transition. | | | | |

## REFERENCES

1. Oviya RP, Gopal G, Shirley SS, Sridevi V, Jayavelu S, Rajkumar T. (2021). Mitochondrial ribosomal small subunit proteins (MRPS) MRPS6 and MRPS23 show dysregulation in breast cancer affecting tumorigenic cellular processes. *Gene*, *790*, 145697. https://doi.org/10.1016/j.gene.2021.145697

2. Lin X, Guo L, Lin X, Wang Y, Zhang G. (2022). Expression and prognosis analysis of mitochondrial ribosomal protein family in breast cancer. *Scientific Reports*, *12*(1), 10658. https://doi.org/10.1038/s41598-022-14724-7

3. Revathi Paramasivam O, Gopisetty G, Subramani J, Thangarajan R. (2021). Expression and affinity purification of recombinant mammalian mitochondrial ribosomal small subunit (MRPS) proteins and protein-protein interaction analysis indicate putative role in tumourigenic cellular processes. *Journal of Biochemistry*, *169*(6), 675-92. https://doi.org/10.1093/jb/mvab004

4. Sotgia F, Fiorillo M, Lisanti MP. (2017). Mitochondrial markers predict recurrence, metastasis and tamoxifen-resistance in breast cancer patients: Early detection of treatment failure with companion diagnostics. *Oncotarget*, *8*(40), 68730-45. https://doi.org/10.18632/oncotarget.19612

5. Ózsvári B, Sotgia F, Lisanti MP. (2020). First-in-class candidate therapeutics that target mitochondria and effectively prevent cancer cell metastasis: mitoriboscins and TPP compounds. *Aging*, *12*(11), 10162-79. https://doi.org/10.18632/aging.103336

6. Li X, Wang M, Li S, Chen Y, Wang M, Wu Z, Sun X, Yao L, Dong H, Song Y, Xu Y. (2021). HIF-1-induced mitochondrial ribosome protein L52: a mechanism for breast cancer cellular adaptation and metastatic initiation in response to hypoxia. *Theranostics*, *11*(15), 7337-59. https://doi.org/10.7150/thno.57804

7. Qi L, Ding Y. (2018). Analysis of metastasis associated signal regulatory network in colorectal cancer. *Biochemical and Biophysical Research Communications*, *501*(1), 113-8. https://doi.org/https://doi.org/10.1016/j.bbrc.2018.04.186

8. Zhou W, Ouyang J, Li J, Liu F, An T, Cheng L, Kuo ZC, Zhang C, He Y. (2021). MRPS17 promotes invasion and metastasis through PI3K/AKT signal pathway and could be potential prognostic marker for gastric cancer. *Journal of Cancer*, *12*(16), 4849-61. https://doi.org/10.7150/jca.55719

9. Chang H, Li J, Qu K, Wan Y, Liu S, Zheng W, Zhang Z, Liu C. (2020). CRIF1 overexpression facilitates tumor growth and metastasis through inducing ROS/NFκB pathway in hepatocellular carcinoma. *Cell Death & Disease*, *11*(5), 332. https://doi.org/10.1038/s41419-020-2528-7

10. Hertweck KL, Vikramdeo KS, Galeas JN, Marbut SM, Pramanik P, Yunus F, Singh S, Singh AP, Dasgupta S. (2023). Clinicopathological significance of unraveling mitochondrial pathway alterations in non‐small‐cell lung cancer. *The FASEB Journal*, *37*(7). https://doi.org/10.1096/fj.202201724rr

11. Dai D, Shi R, Han S, Jin H, Wang X. (2020). Weighted gene coexpression network analysis identifies hub genes related to KRAS mutant lung adenocarcinoma. *Medicine (Baltimore)*, *99*(32), e21478. https://doi.org/10.1097/md.0000000000021478

12. Zhang W, Yu L, Xu C, Tang T, Cao J, Chen L, Pang X, Ren W. (2023). MRPL51 is a downstream target of FOXM1 in promoting the malignant behaviors of lung adenocarcinoma. *Oncology Letters*, *26*(1), 298. https://doi.org/10.3892/ol.2023.13884

13. Liu J, Wu S, Xie X, Wang Z, Lei Q. (2020). Identification of potential crucial genes and key pathways in osteosarcoma. *Hereditas*, *157*(1), 29. https://doi.org/10.1186/s41065-020-00142-0

14. Best CJM, Gillespie JW, Yi Y, Chandramouli GVR, Perlmutter MA, Gathright Y, Erickson HS, Georgevich L, Tangrea MA, Duray PH, GonzáLez S, Velasco A, Linehan WM, Matusik RJ, Price DK, Figg WD, Emmert-Buck MR, Chuaqui RF. (2005). Molecular Alterations in Primary Prostate Cancer after Androgen Ablation Therapy. *Clinical Cancer Research*, *11*(19), 6823-34. https://doi.org/10.1158/1078-0432.ccr-05-0585

15. Kim HJ, Nguyen QK, Jung SN, Lim MA, Oh C, Piao Y, Jin Y, Kim JH, Kim YI, Kang YE, Chang JW, Won HR, Koo BS. (2023). Mitochondrial Ribosomal Protein L14 Promotes Cell Growth and Invasion by Modulating Reactive Oxygen Species in Thyroid Cancer. *Clinical and Experimental Otorhinolaryngol*, *16*(2), 184-97. https://doi.org/10.21053/ceo.2022.01760

16. Xu H, Zou R, Li F, Liu J, Luan N, Wang S, Zhu L. (2021). MRPL15 is a novel prognostic biomarker and therapeutic target for epithelial ovarian cancer. *Cancer Medicine*, *10*(11), 3655-73. https://doi.org/10.1002/cam4.3907

17. Li X-Y, He X-Y, Zhao H, Qi L, Lu J-J. (2023). Identification of a novel therapeutic target for lung cancer: Mitochondrial ribosome protein L9. *Pathology - Research and Practice*, *248*, 154625. https://doi.org/10.1016/j.prp.2023.154625

18. Yuan L, Li JX, Yang Y, Chen Y, Ma TT, Liang S, Bu Y, Yu L, Nan Y. (2021). Depletion of MRPL35 inhibits gastric carcinoma cell proliferation by regulating downstream signaling proteins. *World Journal of Gastroenterology*, *27*(16), 1785-804. https://doi.org/10.3748/wjg.v27.i16.1785

19. Yuan L, Yang Y, Li X, Zhou X, Du YH, Liu WJ, Zhang L, Yu L, Ma TT, Li JX, Chen Y, Nan Y. (2022). 18β-glycyrrhetinic acid regulates mitochondrial ribosomal protein L35-associated apoptosis signaling pathways to inhibit proliferation of gastric carcinoma cells. *World Journal of Gastroenterology*, *28*(22), 2437-56. https://doi.org/10.3748/wjg.v28.i22.2437
